# Spatio-temporal modeling of visceral leishmaniasis in Midwest Brazil: An ecological study of 18-years data (2001–2018)

**DOI:** 10.1371/journal.pone.0240218

**Published:** 2020-10-02

**Authors:** Everton Falcão de Oliveira, Alessandra Gutierrez de Oliveira, Carla Cardozo Pinto de Arruda, Wagner de Souza Fernandes, Márcio José de Medeiros

**Affiliations:** 1 Instituto Integrado de Saúde, Universidade Federal de Mato Grosso do Sul, Campo Grande, MS, Brasil; 2 Programa de Pós-Graduação em Doenças Infecciosas e Parasitárias, Universidade Federal de Mato Grosso do Sul, Campo Grande, MS, Brasil; 3 Instituto de Biociências, Universidade Federal de Mato Grosso do Sul, Campo Grande, MS, Brasil; 4 Campus Macaé, Universidade Federal do Rio de Janeiro, Rio de Janeiro, RJ, Brasil; Faculty of Science, Ain Shams University (ASU), EGYPT

## Abstract

Visceral leishmaniasis (VL) is a neglected vector-borne disease associated with socioeconomic and environmental issues. In Brazil, epidemics of VL have occurred in major cities since 1980. Applied models for medical and epidemiological research have been used to assess the distribution and characteristics of disease endpoints and identify and characterize potential risk factors. This study described the demographic features of VL and modeled the spatio-temporal distribution of human VL cases and their relationship with underlying predicitve factors using generalized additive models. We conducted an ecological study covering an 18-year period from the first report of an autochthonous case of VL in Campo Grande, state of Mato Grosso do Sul, in 2001 to 2018. The urban area of the city has 74 neighborhoods, and they were the units of analysis of our work. Socioeconomic and demographic data available from Brazilian public databases were considered as covariables. A total of 1,855 VL cases were reported during the study period, with an annual mean incidence rate of 13.23 cases per 100,000 population and a cumulative crude incidence of 235.77 per 100,000 population. The results showed the rapid transition from epidemic to endemic and the centrifugal dispersal pattern of the disease. Moreover, the model highlighted that the urban quality of life index, which is calculated based on income, education, housing conditions, and environmental sanitation data, plays a role in VL occurrence. Our findings highlighted the potential for improving spatio-temporal segmentation of control measures and the cost-effectiveness of integrated disease management programs as soon as VL is difficult to control and prevent and has rapid geographical dispersion and increased incidence rates.

## Introduction

Leishmaniases constitute the third group of major importance among vector-borne diseases, with an estimated 1.4 million disability-adjusted life years lost behind only malaria and dengue [1]. Moreover, leishmaniases are considered neglected diseases once they are endemic in low-income populations, with unacceptable morbidity and mortality indicators and reduced investments in research, drug production, and control actions [2, 3].

Visceral leishmaniasis (VL) is the most severe clinical form and is characterized as a chronic and systemic disease that, when left untreated, is lethal in more than 95% of cases [4]. The main etiological agent of VL in Brazil and Latin America is *Leishmania infantum*, whose vectors are *Lutzomyia longipalpis* [5] and *Lutzomyia cruzi* [6, 7] sandflies.

In Brazil, epidemics of VL have been observed in major cities since 1980, when the first evidence of urbanization of the disease was recorded [8, 9]. This continuous increase in incidence in various regions of Brazil may be triggered by environmental changes promoted by rural exodus and other migratory movements, lack of planning and sanitation in urban areas, as well as the adaptation of the vector to domestic reservoirs [5, 10–12]. This context and the territorial spread of the disease represent some of the challenges for disease control in urban areas [13, 14], as observed in the city of Campo Grande, state of Mato Grosso do Sul, where the disease was reported in 2001; it spread rapidly throughout the urban areas of the city and became endemic in a few years [15]. A recent report—which compared the underlying VL risk using a spatio-temporal explicit Bayesian hierarchical model with the risk classification currently in use by Brazil’s Ministry of Health—showed that Campo Grande remains a high-risk area for *L*. *infantum* transmission [16].

Applied models for medical and epidemiological research have been used to assess the distribution and characteristics of disease endpoints and identify and characterize the effect of potential risk factors on these endpoints [17–20]. Understanding the spatial dynamics of the disease and its relationships with socioeconomic and environmental predictors can provide support for the implementation of more effective strategies for the control of infectious diseases [21]. Due to the spatial nature of health events, the application of geostatistical methods is an essential part of the analysis and interpretation of these data [22]. The reason lies in the fact that any data linked to a geographical location may have characteristics associated with its location; that is, the variables may have some location-related correlation structure [22].

Among several methods, generalized additive models (GAMs) [23] were recently used to study the relationship between cutaneous leishmaniasis occurrence and possible risk factors [18] and to predict the potential distribution of *Leishmania* vectors [24]. GAMs are semi-parametric regression methods that relate the response variable to smoothed functions of potential explanatory variables via a link function [23, 25]. Thus, this study aimed to describe the demographic features of human VL and model the spatio-temporal distribution of reported cases of VL using GAM, covering an 18-year period from the first report of an autochthonous case in 2001 to 2018 in an urban area endemic for VL. We also assessed the relationship between the disease and a few underlying predictor factors related to socioeconomic status.

## Materials and methods

### Study area

**Fig 1:**
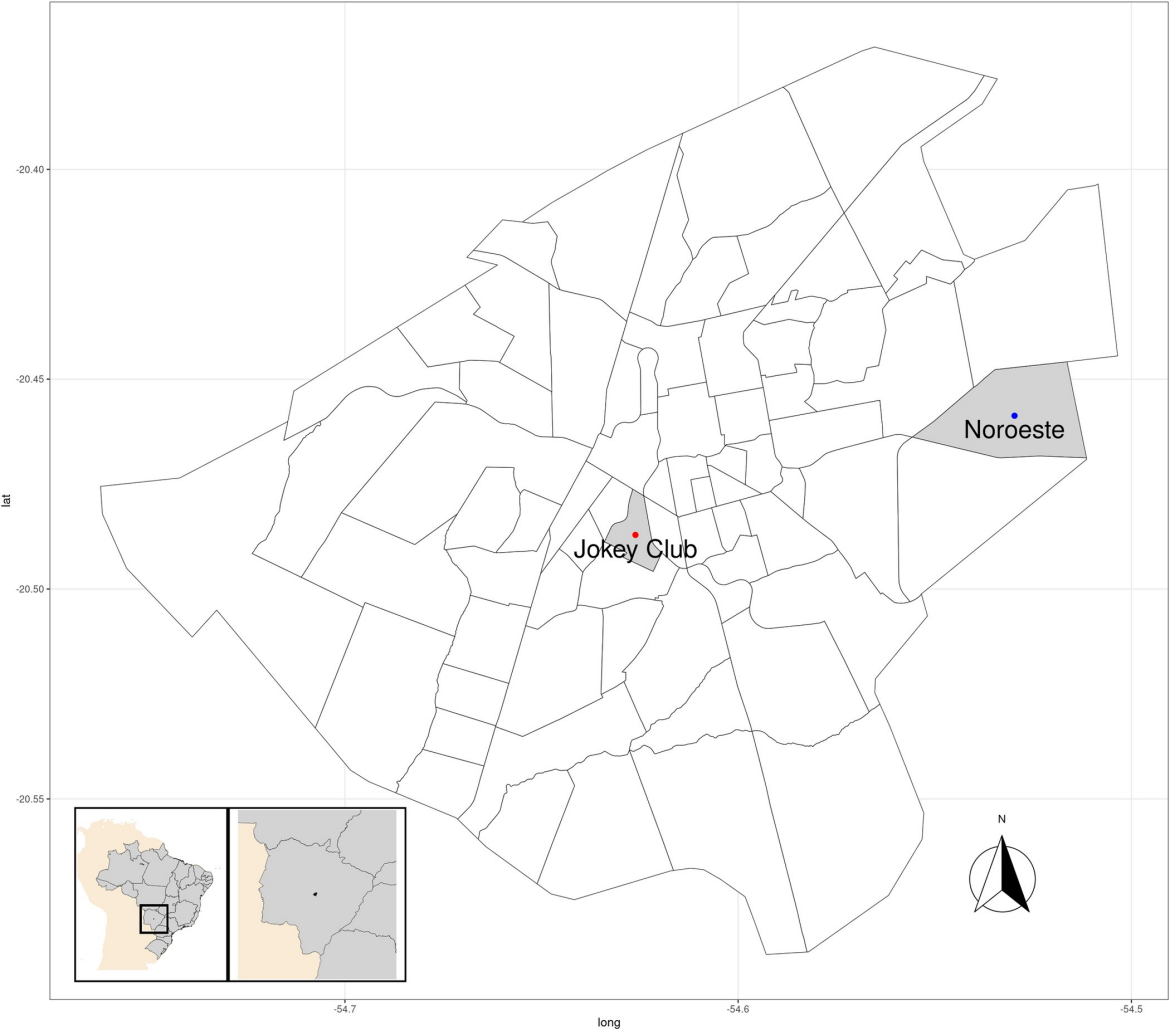
Study area. Jockey and Noroeste neighborhoods are highlighted due to the behavior of their relative smoothed risk distinct from the other neighborhoods, as shown in the results. Data sources: shapefiles from the Brazilian Institute of Geography and Statistics (IBGE) and Municipal Department of Environment and Urban Development of Campo Grande (PLANURB).

Located in the central region of the state of Mato Grosso do Sul, Brazil, Campo Grande (20° 26’ 34” S, 54° 38’ 47” W, Gr) has a total area of 8,118.4 km^2^ (Fig 1), of which the urban area occupies 359.03 km^2^ and is divided into 74 neighborhoods (units of analysis used in this study) [26]. In 2019, according to estimates by the Brazilian Institute of Geography and Statistics (IBGE) [27], Campo Grande had an estimated 895,982 inhabitants. The population density is 97.22 inhabitants/km^2^, and 98.66% of this population lives in urban areas [28]. Moreover, 92.3% of the buildings in Campo Grande are masonry houses with cladding, and 61.5% of the economically active population earns up to two minimum wages. Concerning sanitation, 44%, 90%, and 98.8% of the population has access to sewage treatment, treated water, and garbage collection, respectively [29].

In the Köppen climate classification system, the climate of Campo Grande is tropical monsoon (Am), characterized by irregular rainfall distribution with a well-defined dry season during the coldest months of the year and a rainy season during the summer months [30].

### Study design and data sources

We conducted an ecological study based on reported and autochthonous human cases of VL. The analysis was carried out in two steps: first, the occurrences of the disease were used to calculate the incidence and describe the demographic features. In the second step, the reported cases were geocoded and grouped by neighborhood to estimate the smoothed relative risks and assessed according to the area data analysis using GAM to study the spatio-temporal distribution of the disease.

We considered all confirmed autochthonous human cases of VL reported in the urban perimeter of Campo Grande from January 2001 to December 2018. These data were extracted from the Brazilian Notification Disease Information System (SINAN) [31].

The covariables listed in Table 1 describe the demographic and socioeconomic characteristics of the Campo Grande neighborhoods and were used as covariates to mode the occurrences of LV. These data were extracted from the databases of the IBGE, the Municipal Department of Environment and Urban Planning of Campo Grande [29], and the study Campo Grande social exclusion profile [32]. We have considered in our analysis all variables related to the socioeconomic factors available for the study area. More details and the characterization of these covariables through descriptive measures are presented in the S1 Table.

**Table 1:** Covariates assessed in the study.

Source	Variable
IBGE	
	Number of permanent private households
	Total number of residents per permanent private household
	Average number of residents in permanent private housing units
	Income–value of median monthly nominal income of persons ≥10 years of age
	Proportion of the population with a toilet at home
	Proportion of the population with household water supply
	Proportion of the population with regular garbage collection by a public cleaning service
PLANURB	
	Education index
	Income and poverty index
	Environmental sanitation index
	Housing and living conditions index
	Urban quality of life index
Sauer et al. [32]	
	Social exclusion index
	Poverty of the persons responsible for permanent private housing units
	Income inequality
	Literacy rate
	Years of education of persons responsible for permanent private housing units

Abbreviations: IBGE, Brazilian Institute of Geography and Statistics; PLANURB, Municipal Department of Environment and Urban Planning of Campo Grande.

The grid of the neighborhoods of Campo Grande used in this study was made available in shapefile format (ESRI–Environmental Systems Research Institute) by the Municipal Department of Environment and Urban Planning of Campo Grande.

### Statistical methods

The crude incidence per year and age-sex-specific incidence rates were calculated. In addition, the proportions of notifications by age and sex were calculated per year (available as supplementary data). To compare male and female occurrences by age categories, Poisson regression was used to estimate incidence ratios with 95% confidence intervals. The rates were described using descriptive statistics and presented in the tables and figures.

Considering that the incidence rates do not consider possible differences between the observation units (neighborhoods, in the case of this work), such as the age distribution of individuals and the number of occurrences of VL cases per unit area, the estimate of the relative risk was used for the temporal-spatial analysis. Considering further that the relative risk does not take into account the possible uncertainty associated with unusual incidence rates in counties with relatively small populations at risk [33], the smoothed relative risk (SRR) proposed by Clayton and Kaldor [34] was used to assess the spatial distribution of VL, which allowed us to compare the results between neighborhoods. To estimate the SRR, the observed number of cases was geocoded and grouped by neighborhood, and indirect standardization [35] was used to compute the expected number of cases for each neighborhood. The SRR then follows as the ratio of the observed number of events (reported cases of VL) over the expected number:

$$SRRi=\frac{Oi}{Ei}$$

where *Oi* is the observed or reported number of VL cases in the area (neighborhood) *i*, and *Ei* is the expected number of VL cases for the area *i*.

To assess the relationship between the disease occurrences in the neighborhood and the period investigated with the demographic and socioeconomic variables, we employed a GAM considering the spatio-temporal interactions. According to Wikle, Zammit-Mangion, and Cressie [36], in general, a GAM model considers the transformation of the mean response to have an additive form in which the additive components are smooth functions (e.g., splines) of the covariates, where the functions themselves are generally expressed as basis-function expansions. GAMs can approximate the relationship between the predictors (inputs) and the outcome variable (output) and express the relationship mathematically. The proposed model can be written as the transformed mean response additively as:

g(Y(s;t))=x(s;t)'β+f(s;t)+ν(s;t),

where *Y(s; t)* is the response (SRR or case counts), *g(·)* is a specified monotonic link function, *x(s; t)* is a vector of covariates for spatial location *s* and time *t*, *β* is a vector of parameters, the function *f(s; t)* is a random smooth function of space and time, and *ν(s; t)* is a spatio-temporal white-noise error process; following the notation adopted by Wikle, Zammit-Mangion, and Cressie [36].

To avoid the effects of multicollinearity, at the beginning of the modeling process, the correlations were assessed using the Pearson correlation coefficient, and one of the covariables between the pairs with a correlation greater than 0.8 was excluded. After the adjustment, the correlations between the estimated coefficients were verified, excluding the covariables with a correlation between coefficients greater than 0.7 as suggested by Seber and Lee [37]. Then, the stepwise backward method (p-value < 0.05) was adopted to select the model's explanatory variables [38]. In the last step, cross-validation was adopted to define the parameters of the time-space effect (node parameters). Data from 2018 were not included in the estimation process; they were used only in the cross-validation process, that is, the mo

del estimated with data from 2001–2017 was used to predict the year 2018, with the model that presented the lowest mean squared error chosen as the final model. This process was repeated to adjust the soft risk (with gamma response) and occurrences (with Poisson and negative binomial response) [39]. The residues were checked to assess whether the model adequately captured the spatial and temporal variability in the data. Considering Henebry’s approach [40], Moran’s *I* test was used to test the spatial dependence, and the Durbin-Watson test was used for temporal dependency [41].

Statistical analyses of the data, generation of the maps, and modeling were performed using R 3.6.1. The ggmap package [42] was used to perform the geocoding, and ggplot2 [43] was used to plot the maps. The smooth relative risks were estimated using the Dcluster package [44], and the binomial negative GAM was estimated using the mgcv package [45]. The ape [46] and lmtest [47] packages were used respectively for the Moran’s *I* and Durbin-Watson tests. Scatter plots and matrix correlations were built using the PerformanceAnalytics package [48].

### Ethics statement

This study was approved by the Research Ethics Committee of the Federal University of Mato Grosso do Sul (CAAE: 02617218.8.0000.0021) and registered under number 3.030.880. Personally identifiable information (patient name and information included on the case report form) was available only to surveillance officers and was not used in this study.

## Results

From 2001 to 2018, a total of 1,855 cases of VL were reported in Campo Grande, with an annual average incidence rate of 13.23 cases per 100,000 population and a cumulative crude incidence of 235.77 per 100,000 population for the period. The distribution of cases by sex and age group is shown in Tables 2 and 3. Regarding age, children between 0 and 5 years and adults over 40 years of age were the most affected by the disease. It is noteworthy that since the beginning of the epidemic in 2001, children had a high risk of illness. Regarding sex, in general, the highest incidence was recorded in men. When analyzing sex and age, although the incidence of VL in men was higher in almost all age groups, no statistical difference was observed when the male-to-female incidence rate ratio (IRR) was estimated overall or stratified by age group (IRR: 1.92; 95% confidence interval [CI]: 0.53–6.90). During the 18 years evaluated, the male/female ratio remained practically constant during the first five years of the epidemic, it oscillated with little variability between 2006 and 2016 and returned to the initial ratio in the final two years of the analysis.

**Table 2:** Demographic features of visceral leishmaniasis cases in Campo Grande, Brazil, 2001–2018.

**Age**	**2001**		**2002**		**2003**		**2004**		**2005**		**2006**		**2007**		**2008**		**2009**		**2010**	
	n	%	n	%	n	%	n	%	n	%	n	%	n	%	n	%	n	%	n	%
< 1	0	0,00	0	0,00	2	2,08	9	7,14	13	8,50	11	6,96	11	8,27	14	9,79	7	6,80	5	4,59
1 to 4	5	50,00	6	30,00	25	26,04	30	23,81	27	17,65	34	21,52	26	19,55	30	20,98	21	20,39	25	22,94
5 to 14	1	10,00	5	25,00	17	17,71	20	15,87	32	20,92	20	12,66	21	15,79	16	11,19	8	7,77	12	11,01
15 to 24	2	20,00	3	15,00	16	16,67	14	11,11	20	13,07	15	9,49	17	12,78	12	8,39	8	7,77	10	9,17
25 to 39	1	10,00	3	15,00	11	11,46	22	17,46	23	15,03	32	20,25	18	13,53	18	12,59	14	13,59	15	13,76
40 to 59	0	0,00	2	10,00	17	17,71	22	17,46	24	15,69	28	17,72	29	21,80	36	25,17	28	27,18	28	25,69
≥ 60	1	10,00	1	5,00	8	8,33	9	7,14	14	9,15	18	11,39	11	8,27	17	11,89	17	16,50	14	12,84
Total	10		20		96		126		153		158		133		143		103		109	
**Sex**																				
F	4	40,00	8	40,00	39	40,63	50	39,68	57	37,25	44	27,85	45	33,58	50	34,97	33	32,04	48	43,64
M	6	60,00	12	60,00	57	59,38	76	60,32	96	62,75	114	72,15	89	66,42	93	65,03	70	67,96	62	56,36
Total	10		20		96		126		153		158		134		143		103		110	
**Age**	**2011**		**2012**		**2013**		**2014**		**2015**		**2016**		**2017**		**2018**		**Total**			
	n	%	n	%	n	%	n	%	n	%	n	%	n	%	n	%	n	%		
< 1	16	11,43	9	4,48	6	3,85	4	4,55	8	11,59	2	3,70	2	3,33	3	8,82	122	6,58		
1 to 4	29	20,71	52	25,87	20	12,82	12	13,64	13	18,84	7	12,96	11	18,33	6	17,65	379	20,45		
5 to 14	15	10,71	9	4,48	8	5,13	7	7,95	2	2,90	1	1,85	2	3,33	3	8,82	199	10,74		
15 to 24	8	5,71	17	8,46	10	6,41	14	15,91	2	2,90	2	3,70	2	3,33	1	2,94	173	9,34		
25 to 39	19	13,57	30	14,93	33	21,15	13	14,77	19	27,54	22	40,74	10	16,67	10	29,41	313	16,89		
40 to 59	35	25,00	51	25,37	48	30,77	17	19,32	16	23,19	15	27,78	20	33,33	8	23,53	424	22,88		
≥ 60	18	12,86	33	16,42	31	19,87	21	23,86	9	13,04	5	9,26	13	21,67	3	8,82	243	13,11		
Total	140		201		156		88		69		54		60		34		1853			
**Sex**																				
F	52	37,14	66	32,84	57	36,77	28	31,82	28	40,58	14	25,93	24	40,00	13	38,24	660	35,60		
M	88	62,86	135	67,16	98	63,23	60	68,18	41	59,42	40	74,07	36	60,00	21	61,76	1194	64,40		
Total	140		201		155		88		69		54		60		34		1854			

Note: One case was excluded from sex analysis and two from age analysis because of missing information.

**Table 3:** Cumulative crude incidence of visceral leishmaniasis according to age and sex; Campo Grande, Brazil, 2001–2018.

	Female sex			Male sex			Male-to-female Incidence rate ratio	(95% CI)
	Population	Cases	Incidence per 100,000	Population	Cases	Incidence per 100,000		
**Age**	405464	660	162,78	381333	1192	312,59	1.92	0.53–6.90
< 1	5734	53	924,31	5965	69	1.156,75	1.25	0.81–1.94
1 to 4	22153	187	844,13	23109	192	830,85	0.98	0.95–1.02
5 to 9	27542	62	225,11	28829	76	263,62	1.17	0.86–1.60
10 to 14	31843	33	103,63	32845	28	85,25	0.82	0.56–1.21
15 to 19	35218	27	76,67	35337	44	124,52	1.62	0.63–4.20
20 a 29	73723	68	92,24	73142	125	170,90	1.85	0.55–6.21
30 a 39	66420	65	97,86	61218	156	254,83	2.60	0.40–16.99
40 a 49	57372	67	116,78	50412	187	370,94	3.18	0.33–30. 60
50 a 59	41698	29	69,55	36006	141	391,60	5.63	0.19–166.58
60 a 69	24028	39	162,31	19980	88	440,44	2.71	0.38–19.20
70 a 79	13496	21	155,60	10282	58	564,09	3.63	0.29–45.25
≥ 80	6237	9	144,30	4208	28	665,40	4.61	0.23–92.23

Note: One case was excluded from sex analysis and two from age analysis because of missing information.

CI, confidence interval.

**Fig 2:**
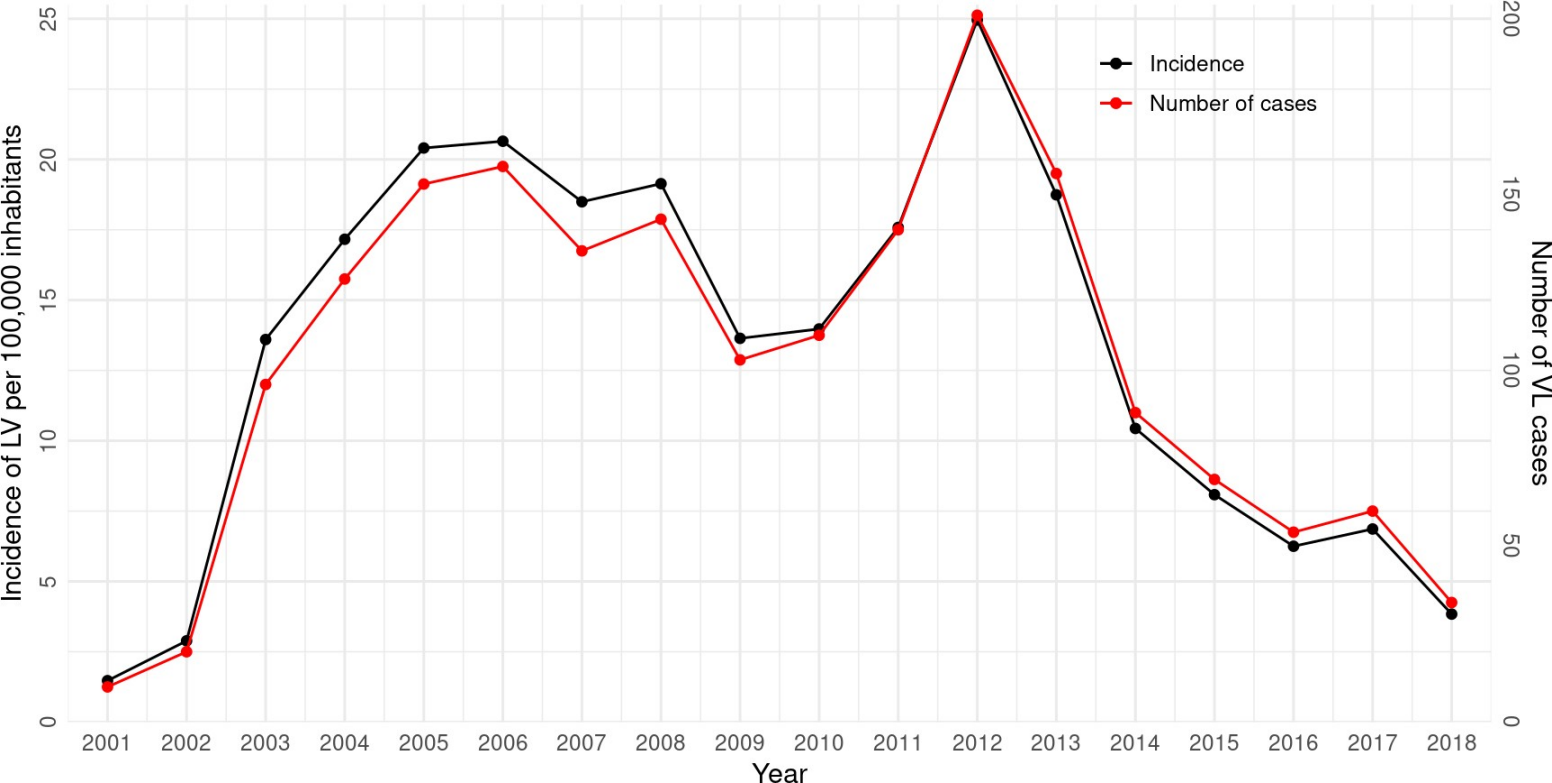
Annual crude incidence and absolute frequency of visceral leishmaniasis by year in Campo Grande, Brazil, 2001–2018.

The annual crude incidence and the temporal evolution of VL cases are depicted in Fig 2. Descriptively, there was a continuous and progressive increase in the incidence rate until 2006, followed by declines in 2007, 2009, and 2010, and a sharp increase between 2011 and 2012. From 2013 through 2018, the tendency was for the incidence to decrease.

**Fig 3:**
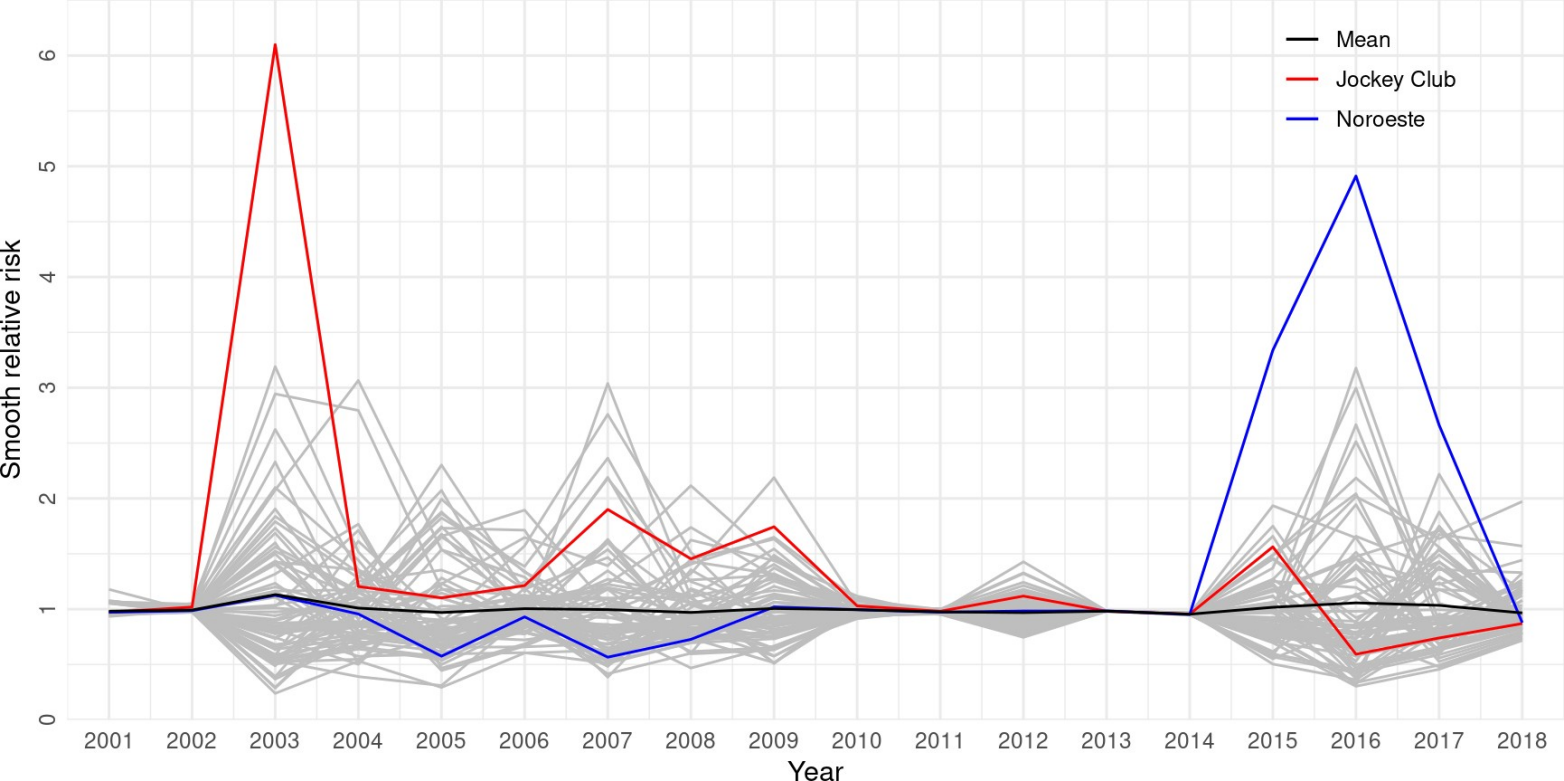
Smoothed relative risks according to the neighborhoods of Campo Grande, Brazil, 2001–2018 (n = 1840).

**Fig 4:**
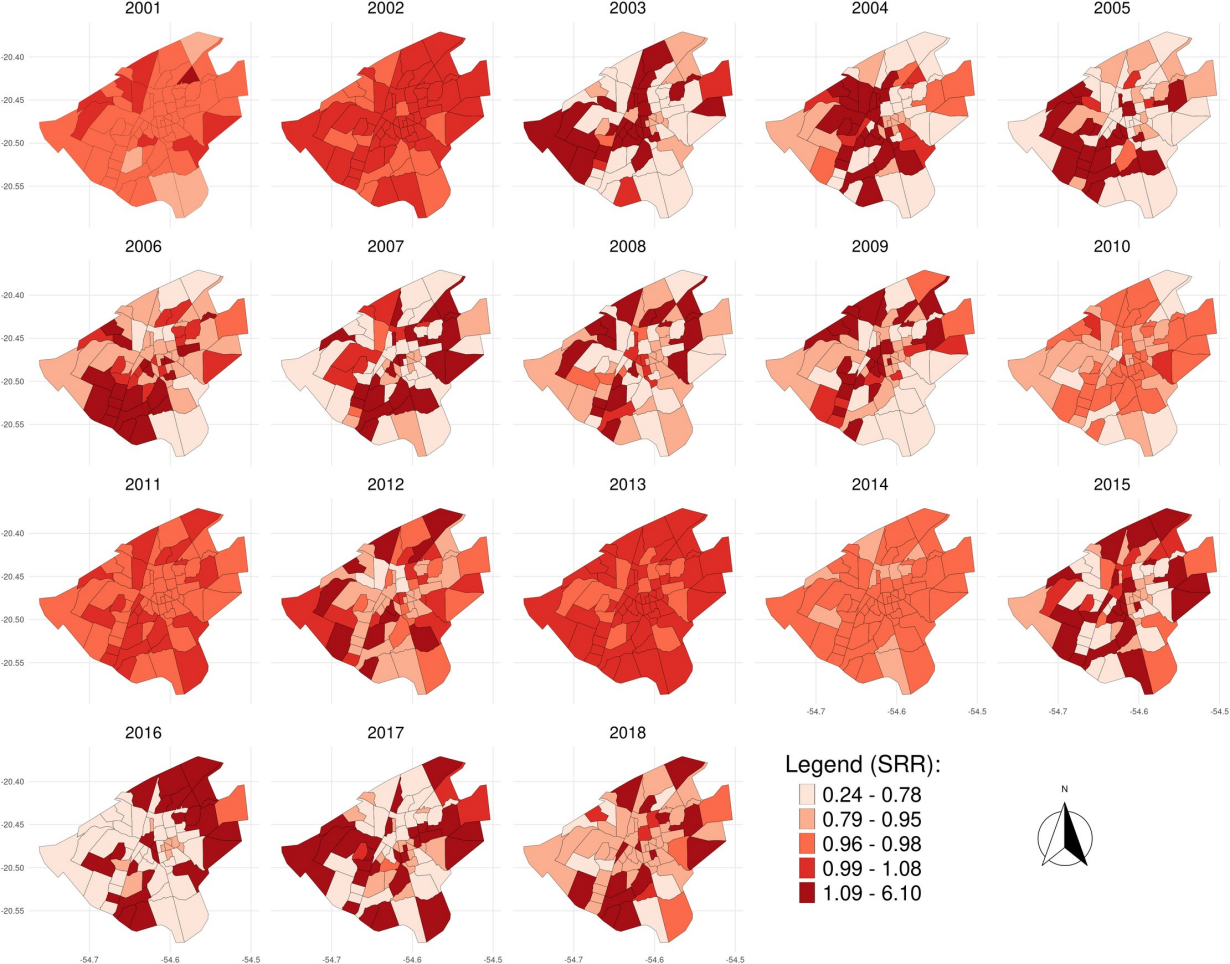
Spatial distribution of smoothed relative risks according to year and neighborhoods; Campo Grande, Brazil, 2001–2018. Legend categories coded with the light red tones represent neighborhoods where the risk is less than the city average (SRR < 1), and the dark red tones corresponding to those neighborhoods where the risk is higher than the city average (SRR > 1). Data sources: shapefile from the Municipal Department of Environment and Urban Development of Campo Grande (PLANURB); Brazilian Notification Disease Information System (SINAN). Geographic Coordinate Systems WGS-84. SRR, smoothed relative risk.

Fig 3 shows the smoothed relative risks for each Campo Grande neighborhood throughout the evaluated series. Among the 1,855 notifications, 15 cases who lived in the rural area when they were diagnosed and reported to the SINAN were excluded from the analysis. Descriptively, it is noted that there was a relatively high fluctuation (variability) until 2010, followed by stabilization between 2010 and 2014, with a return to baseline from 2014. Two neighborhoods showed different behaviors and, therefore, improved detail was required: at the beginning of the series, in 2003, the Jockey Club neighborhood had a high SRR that decreased over time; the Noroeste neighborhood, on the other hand, showed the opposite behavior and was conspicuous due to the sharp increase in rates between 2014 and 2016, peaking in 2016. The explanation for the high rates is that the observed values were much higher than the expected values for these areas. Fig 4 presents the spatial distribution of SRR according to neighborhoods over the study period.

**Fig 5:**
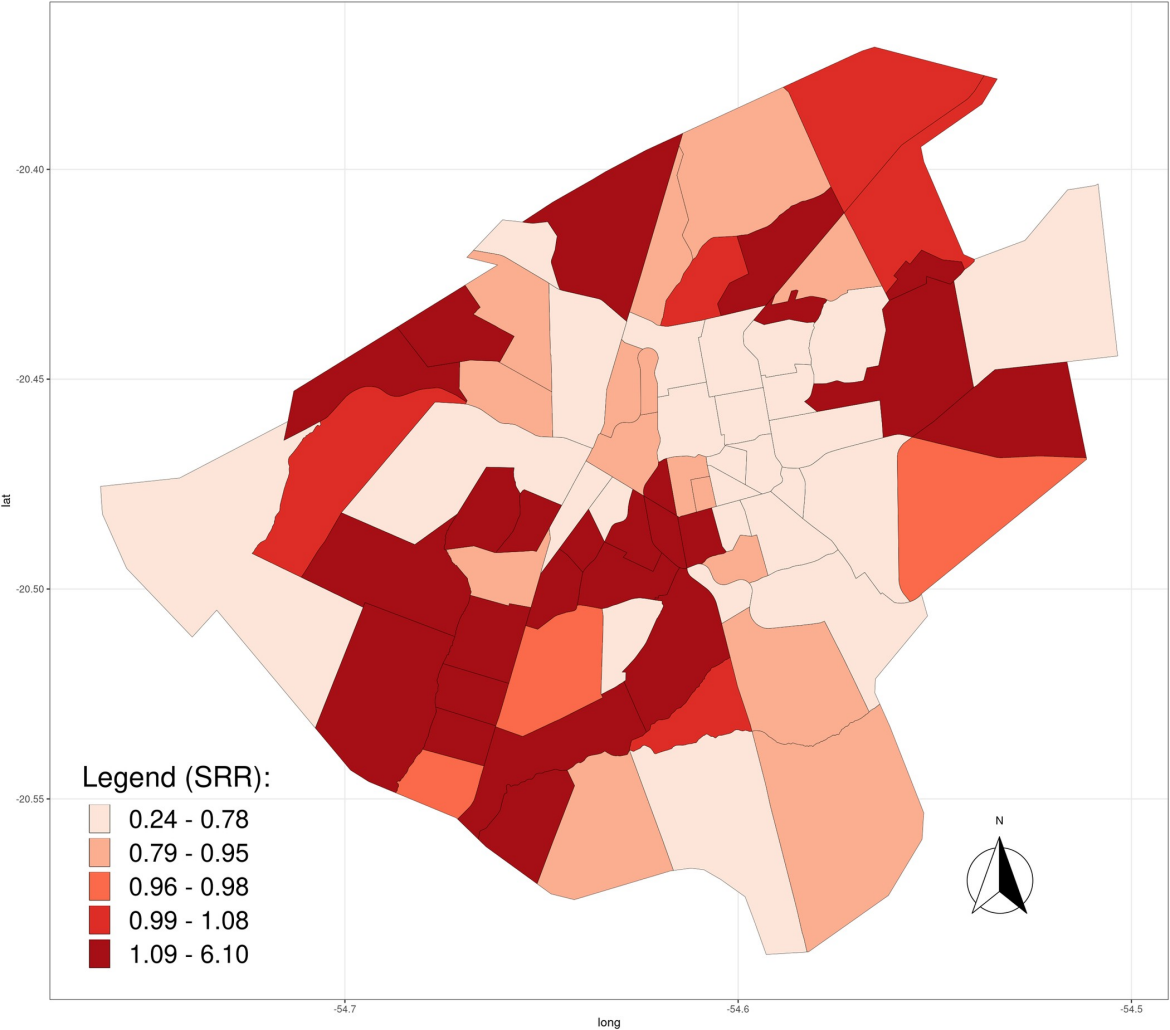
Spatial distribution of cumulative smoothed relative risks according to neighborhoods in Campo Grande, Brazil, 2001–2018. Legend categories coded with the light red tones represent neighborhoods where the risk is less than the city average (SRR < 1), and the dark red tones correspond to those neighborhoods where the risk is higher than the city average (SRR > 1). Data sources: shapefile from the Municipal Department of Environment and Urban Development of Campo Grande (PLANURB); Brazilian Notification Disease Information System (SINAN). Geographic Coordinate Systems WGS-84. SRR, smoothed relative risk.

At the beginning of the VL epidemic, between 2001 and 2003, in addition to the continuous increase in annual incidence rates, there was also a rapid spread of the disease throughout the city that evidenced the transition from epidemic to endemic in Campo Grande in the following years, since the constant presence of the disease was observed in the city. From Fig 4 it can be seen that the high SRR values are distributed throughout the city over the years, and the number of neighborhoods coded with dark red tones (SRR > 1) has also increased over the years, especially after 2003. Considering the spatial distribution of the SRR accumulated in the period 2001–2018 (Fig 5), it was observed that the largest SRRs are distributed in peripheral neighborhoods that, in the great majority, are neighborhoods with low socioeconomic status.

**Fig 6:**
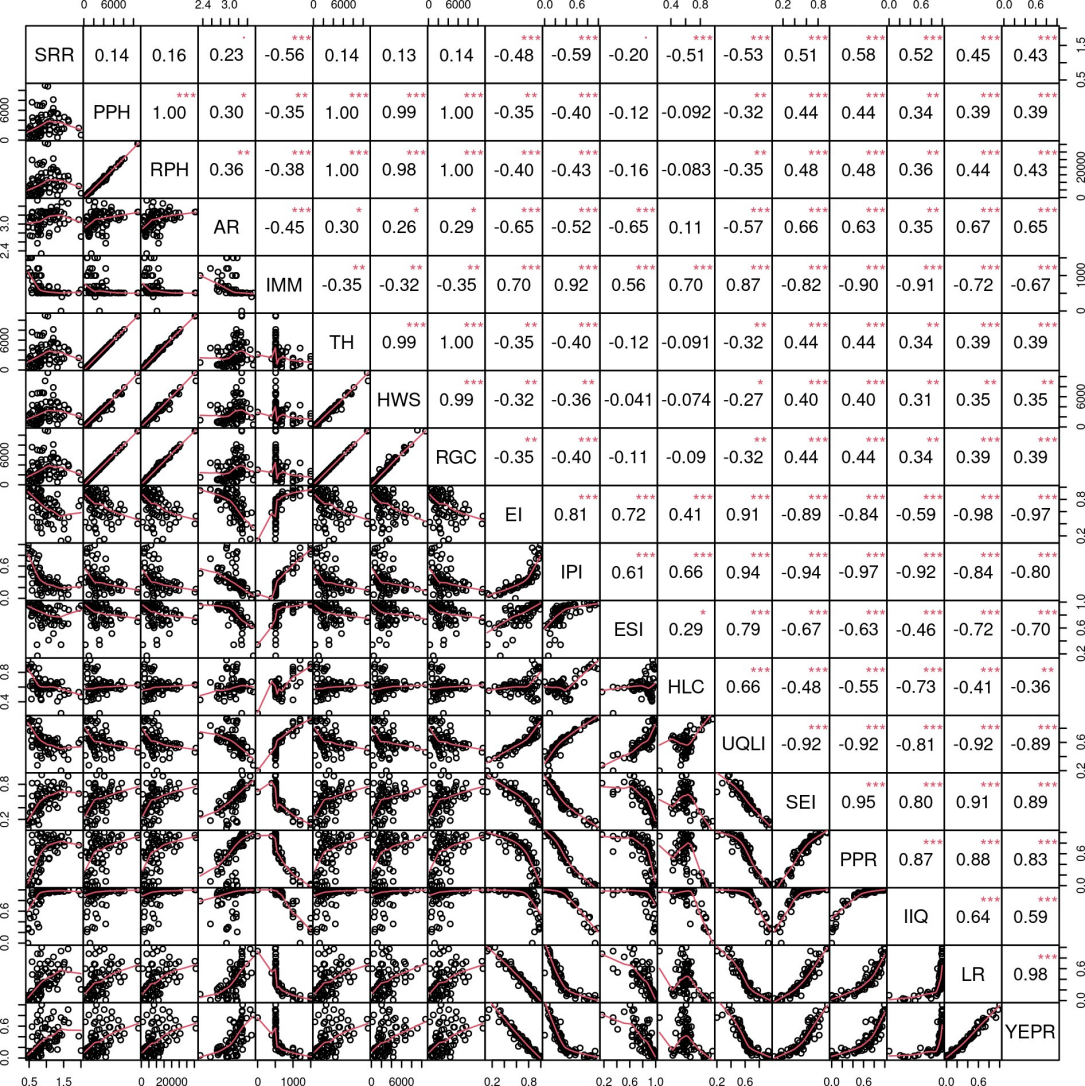
Scatter plots and matrix correlations of the study variables assessed. *** indicates p-value < 0.001; ** indicates p-value < 0.05; * indicates p-value = 0.10; SRR, smoothed relative risk of visceral leishmaniasis; PPH, permanent private households; RPH, residents private household; AR, average number of residents in permanent private housing units; IMM, income value of median monthly nominal income of persons 10 years of age and over; TH, toilet at home; HWS, household water supply; RGC, regular garbage collection; EI, education index; IPI, income and poverty index; ESI, environmental sanitation index, HLC, housing and living conditions index; UQLI, urban quality of life index; SEI, social exclusion index; PPR, poverty of the persons responsible for permanent private housing units; IIQ, income inequality; LR, literacy rate; YEPR, years of education of persons responsible for permanent private housing units.

Our results from Fig 6 and S2 Table showed that among 17 covariables assessed, 10 of them showed a significant association with the cumulative SRR, being that all of them are related to income, housing, or education. The significant correlations between the SRR and the covariables can be considered moderate, since they are around 0.50, with the highest correlation coefficient equal to -0.59 (p-value < 0.001) which corresponded to the *income and poverty index* variable. The second highest correlation coefficient had the opposite direction (*r* = 0.58; p-value < 0.001) and corresponded to the *poverty of the persons responsible for permanent private housing units* variable. These two covariables express the same magnitude (income) and are, therefore, strongly correlated (*r* = -0.92; p-value < 0.001). However, it is important to note that they indicate opposite directions (ratified by the *r* = -0.92; p-value < 0.001), that is, while high values of *income and poverty index* indicate higher income, high values of *poverty of the persons responsible for permanent private housing units* indicate greater poverty among persons responsible for permanent private housing. Moreover, the correlations of these two covariables with the SRR are consistent as they indicate that the higher the poverty level, the greater the SRR. These results can also be viewed in the scatter plots (Fig 6) where weak/moderate linear relationships with apparent non-constant variability are shown; thus, it can be concluded that the SRR of VL has a weak or moderate linear relationship with all covariables. The strong linear relationship between the covariables indicates multicollinearity.

**Fig 7:**
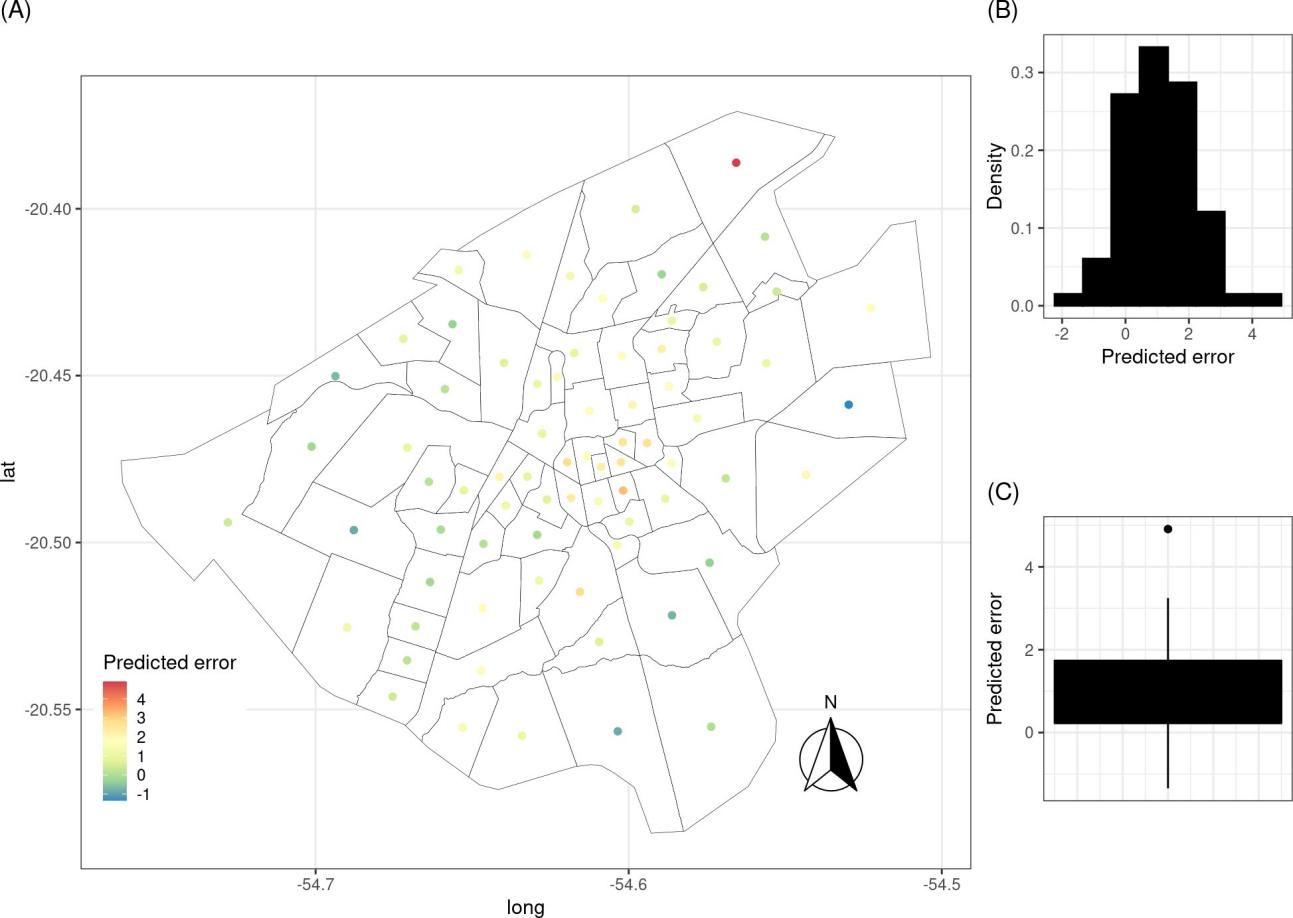
Model predicted errors for 2018. (A) Distribution of the prediction error by the adjusted model of the VL cases in Campo Grande for 2018. (B) Histogram of prediction errors. (C) Boxplot of prediction errors. VL, visceral leishmaniasis.

A GAM with a negative binomial response for the number of VL reports adequately described the trend of VL over the evaluated period. Among the study covariables, only urban quality of life index (UQLI) remained as a predicted variable in the model. The estimated parameters of the model are presented in Table 4. This model presents residuals with Moran’s *I* index and Durbin-Watson test not significant, which indicates that the model adequately captured the spatial and temporal variability in the data. Additionally, the predicted errors for 2018 (Fig 7) reinforced the good model fit, since most predicted errors (difference between the values observed for 2018 and the prediction for this same year) are around 0.

**Table 4:** Parametric coefficients of the GAM regression model with a negative binomial response for the number of VL reports.

	Estimate	Std. Error	z value	Pr(>|t|)
Intercept	-7.9251	0.1827	-43.383	<0.001
UQLI	-2.0471	0.3076	-6.656	<0.001

Abbreviations: GAM, generalized additive model; VL, visceral leishmaniasis; UQLI, urban quality of life index.

## Discussion

In our study, the analysis of an 18-year series in an endemic urban area considered an area of intense VL transmission [16, 49] revealed important findings regarding the epidemiology and spatio-temporal distribution of the disease by highlighting the rapid transition from epidemic to endemic status. This analysis has also indicated a greater occurrence of diseases in extremes of age and an inverse association with covariables related to socioeconomic status, suggesting the greatest risk of illness in vulnerable human populations [50].

Our results indicated that the disease had a heterogeneous incidence in the population, affecting mainly men and extremes of age. Previous studies on the epidemiological profile of VL morbidity and mortality between 2001 and 2009 in the city of Campo Grande [15, 51–53] revealed that men were significantly more affected by the disease than women. The highest morbidity and mortality measures observed among men were associated with age, increasing in individuals over 40 years old and children under 10 years old. In the city of Natal, state of Rio Grande do Norte, Lima et al. [54] reported that the average age at diagnosis increased over prior years, and males were more frequently affected between 1990 and 2014. Some authors have suggested that the immunologic effects of sex hormones could be linked to the increased risk of VL in males [54, 55]. Other studies conducted in urban areas in Brazil have described the higher incidence of the disease in children under 5 years of age, suggesting that this is possibly related to increased susceptibility to *L*. *infantum* infection when long-term immunity is developing [56, 57]. Similar reasoning can be applied for older people, whose other chronic degenerative morbidities and immunosenescence [58] may increase susceptibility to infection.

During the 18-years of VL occurrence in Campo Grande, it was possible to observe variations in the incidence of the disease in two periods, 2008–2009 and 2013–2016. These reductions in incidence rates probably do not have a straightforward explanation, especially due to the complexity of *Leishmania* parasite transmission dynamics [59]. Several factors and hypotheses can be considered, including the cyclical nature of the disease, aspects of its pathogenesis such as undetermined incubation period and asymptomatic and subclinical forms [60, 61], and the discontinuity of control measures recommended by Brazil’s Ministry of Health [16] such as the euthanasia of seropositive dogs, the monitoring of vectors, and the sprinkling of residual action insecticides. From 2007 to 2009, dogs were fitted with a 4% deltamethrin-impregnated collar on a large scale [15]. Data reported by Brazuna [15] showed a reduction in the incidence of canine VL during the two years of this intervention. Although there are no data on the effect of canine VL on the incidence of the disease in humans, our results (Fig 2) showed that the period of high collar coverage in dogs (2008–2009) coincided with a reduction of human cases.

The rapid VL spatio-temporal dispersion and its association with UQLI, which is calculated from socioeconomic and environmental data, are pronounced. The first evidence of the spread and urbanization of VL in Brazil was described by Deane [62] in 1956, in Sobral, in the state of Ceará, Northeast Brazil. Almost 30 years later, the first major urban VL epidemic in Brazil was reported in Teresina, capital of the state of Piauí, also located in the Northeast region [8]. Antonialli et al. [63] suggested that the expansion of VL in the state of Mato Grosso do Sul occurred from the city of Corumbá and coincided in time and space with three major works that would have caused anthropogenic environmental changes, especially the Brazil-Bolivia gas pipeline.

In Campo Grande, our data showed that the disease is associated with covariables related to socioeconomic status. The influence of socioeconomic factors on VL has been widely reported in the scientific literature [11, 64, 65]. The link between poverty and health problems is complex and profound; various conditions are associated with poverty, such as malnutrition, poor housing conditions, difficulties in accessing health services, and a lack of education [65].

The burden of leishmaniasis falls disproportionately on the poorest segments of the global population. In endemic areas, there is an increased risk of infection due to poor housing conditions and environmental sanitation and also due to migratory movements motivated by different causes [64, 66] that favor exposure and contact of non-immune individuals with infected vectors. However, within poor communities, low income may not be a major determinant of risk [64]. In our study, income, education, and housing were inversely associated with VL. The final model with the best fit to the data to explain the occurrence of the disease in the period evaluated in Campo Grande was composed only by UQLI. However, this single predicted variable jointly reflects income, education, housing conditions, and environmental sanitation.

During a major urban VL epidemic in Teresina, Piauí, from 1993 to 1996, the cases were clustered on the outskirts of the city in areas bordering forest and green pastures, in regions with no sewage system [20, 67]. Analyses of this epidemic by multilevel modeling showed that the incidence of the disease was associated with low socioeconomic status, the presence of dense vegetation, and a high prevalence of canine infection [67]. Other studies conducted in Teresina during 1991–2000 [68] and 2001–2006 [69] also reported the spatial correlation of VL incidence rates with socioeconomic, demographic, and risk indicators as well as environmental sanitation such as the presence of running water, suggesting that the occurrence of the disease is associated with poor living conditions.

Our results did not demonstrate the isolated association of VL with indicators of basic and environmental sanitation, such as garbage collection, sanitary sewage, and running water. According to data from the Municipality of Campo Grande in 2012, the public water supply system served 99.5% of the population, and the city’s sewage system with collection and treatment was available to 64.73% of households [29]. However, there are areas in the city with deficiencies in sanitary infrastructure with poor housing conditions. Some of these conditions, such as peridomicile rich in organic matter from fruit trees and household waste, are favorable for the proliferation of vector insects [70, 71].

The absence of a cause-and-effect relationship between demographic density and disease presence suggests the influence of other elements on the maintenance of endemicity in a given area [72]. The present study did not consider the biotic and abiotic environmental factors that are known to be associated with the risk of *L*. *infantum* infection. These factors directly affect the presence, behavior, and distribution of wild parasite vectors and reservoirs and may provide contact with these peridomiciliary areas. Similar to other urban centers, dogs are the main reservoir of *L*. *infantum* in Campo Grande, where serological positivity reached 25% of the total samples analyzed between 2002 and 2006 [52].

Negative binomial Bayesian geostatistical models used to analyze the incidence of leishmaniases in Brazil, which considered climate, environmental, and socioeconomic variables as predictors, demonstrated that rainfall and socioeconomic variables were risk factors for cutaneous and visceral leishmaniases [19]. In Bihar, India, rainfall, illiteracy rate [73, 74], housing type, number of informal workers [71], land use and cover, vegetation conditions, surface humidity, indoor climate, and size of the unemployed population [72] were factors associated with disease occurrence. However, it must be noted that in India, the vector and *Leishmania* are of different species. In this South Asian nation, humans are the reservoir of *L*. *donovani* responsible for anthroponotic VL transmission, which may present a different scenario for VL dispersion from that in Latin America [50].

The rapid and sometimes disorganized Brazilian territorial expansion and urbanization of the disease bring to discussion the control and management strategies advocated by the competent agencies, especially in urban centers where problems of malnutrition, education, housing, and basic sanitation are present [11]. The elimination of VL in Latin America does not seem to be a realistic goal at this time, given the lack of political commitment, gaps in scientific knowledge, and the weakness of management processes and surveillance systems [13, 14]. Thus, the need for studies with improved methodological quality in new regions is evident, prioritizing investigation into the identified patterns and their causes as well as the variables for which knowledge is scarce [75].

This study identified the need to investigate and analyze the association between VL and other predicted variables through more complex and robust models and, perhaps, the incorporation of other climate and environmental variables capable of highlighting the effect of other factors on the spatio-temporal dynamics of the disease. Despite the flexibility of our model that provided a better assumption of the nature of relationships between the UQLI and VL cases, this limitation has to be pointed out.

Even though it is not possible to establish causal inferences from ecological studies, they allow the analysis of certain questions due to the evaluation of the association of a certain disease and variables of interest that are defined in aggregates of individuals [76, 77]. Studies have helped us to understand some factors related to the dynamics of VL dissemination in Brazilian cities, a phenomenon that was poorly understood until the early 1990s [19, 20, 49, 78, 79].

Spatio-temporal models are useful for studying the interrelationships between health, environmental, and socioeconomic factors, as well as the temporal and spatial distribution of various diseases. These studies have provided important information for health surveillance, such as monitoring and mapping of public health impact risk factors, as well as allowing a better description, understanding, and prediction of risk areas for different diseases [67, 80–82]. Particularly, GAM models showed better fit and good prediction accuracy when compared to generalized linear models, which supports the use of this technique in the field of epidemiology where a causal link needs to be assessed [25]. The practical use of this method has been demonstrated through a real data analysis [18, 25].

In conclusion, our manuscript showed that VL has a higher incidence in men and people of extreme ages. About two years after the first autochthonous reported VL case, the disease had already been reported in almost every neighborhood of Campo Grande. The spatio-temporal model presented a good fit to the study data and showed the relationship of the disease as an indicator of urban quality of life, which is related to income, education, housing, and environmental sanitation. These variables were not included individually in the final model, which reinforces the need for a composite index that summarizes the main dimensions of the socioeconomic context for research purposes, considering that countless factors of different scales and dimensions may have interplay with each other [83]. Finally, our results demonstrate the need for investments in integrated control measures that aim beyond the public health measures and policy already recommended by the Ministry of Health of Brazil for VL [16], such as improvements in housing conditions, environmental sanitation, and access to health services, to reduce health disparities observed in this scenario.

## Supporting information

S1 Table Descriptive measures of covariables assessed in the study. Abbreviations: IBGE, Brazilian Institute of Geography and Statistics; PLANURB, Municipal Department of Environment and Urban Planning of Campo Grande.
(PDF)

S2 Table Correlation matrix of smooth relative risk and covariables. (XLS)
