# Supplementary material for: Spatio-temporal modeling of visceral leishmaniasis in Midwest Brazil: An ecological study of 18-years data (2001–2018)
Source: PLoS One. 2020 Oct 2;15(10):e0240218. doi: 10.1371/journal.pone.0240218 (PMC7531797; doi:10.1371/journal.pone.0240218)
Supplement: S1 Table — Abbreviations: IBGE, Brazilian Institute of Geography and Statistics; PLANURB, Municipal Department of Environment and Urban Planning of Campo Grande. (PDF) [file pone.0240218.s001.pdf]

Supporting Information

S1 Table. Descriptive measures of predictor/explanatory variables assessed in the study.

Source	Variable	Mean	Median	Standard deviation	Minimum	Maximum
IBGE						
	Number of permanent private households	3,321.20	2,635	2,314.69	305.00	11,005.00
	Total number of residents per permanent private household	10,382.04	8,242	7,518.75	942.00	36,011.00
	Average number of residents in permanent private housing units	3.08	3.11	0.22	2.34	3.53
	Income – value of median monthly nominal income of persons ≥10 years of age	666.64	510	288.87	0.00	1,500.00
	Proportion of the population with a toilet at home	3318.66	2,631	2,313.16	305.00	10,998.00
	Proportion of the population with household water supply	3,041.51	2,455	2,143.11	198.00	10,762.00
	Proportion of the population with regular garbage collection by a public cleaning service	3,312.89	2,633.50	2,311.06	280.00	11,002.00
PLANURB						
	Education index – reflects the educational situation of the population and was calculated from:	0.68	0.72	0.21	0.12	0.99

	• % of heads of permanent private housing units with ≥1 year of schooling;<br>• % of literate people >15 years of age;<br>• Average number of years of education of persons responsible for permanent private housing units;<br>• Literacy rate of individuals aged 15 to 24 years.					
	Income and poverty index – reflects the economic situation of the population income inequality levels and was calculated from:<br>• % of heads of permanent private housing units with a nominal monthly income higher than 01 minimum wage;<br>• % of heads of permanent private housing units with monthly nominal income;<br>• Average monthly nominal income of persons in charge of permanent private housing units.	0.35	0.31	0.23	0.00	0.99
	Environmental sanitation index – reflects urban services infrastructure and was calculated from the proportions of households with:<br>• A regular garbage collection service;<br>• Piped drinking water;<br>• Sewage pipes;<br>• Bathroom or toilet.	0.80	0.86	0.17	0.17	0.99
	Housing and living conditions index – reflects housing conditions and was calculated from:<br>• % of own permanent private housing units built on own land;	0.62	0.60	0.12	0.24	0.97

	• % of persons residing in permanent private housing units;<br>• Average number of bathrooms for exclusive use of residents in these households.					
	Urban Quality of Life Index – reflects urban quality of life in terms of the four indices listed above.	0.61	0.59	0.16	0.20	0.95
SAUER et al., 2012						
	Social exclusion index	0.53	0.55	0.21	0.05	0.96
	Poverty of the persons responsible for permanent private housing units	0.62	0.71	0.30	0.00	1.00
	Income inequality	0.87	0.97	0.22	0.00	1.00
	Literacy rate	0.39	0.34	0.26	0.00	1.00
	Years of education of persons responsible for permanent private housing units	0.35	0.32	0.26	0.00	1.00

Abbreviations: IBGE, Brazilian Institute of Geography and Statistics; PLANURB, Municipal Department of Environment and Urban Planning of Campo Grande.
